# Languishing in the crossroad? A scoping review of intersectional inequalities in mental health

**DOI:** 10.1186/s12939-019-1012-4

**Published:** 2019-07-24

**Authors:** Nadja Fagrell Trygg, Per E. Gustafsson, Anna Månsdotter

**Affiliations:** Department of Epidemiology and Global Health, Umeå University, Umeå, Sweden

**Keywords:** Intersectionality, Methods, Health inequalities, Mental health and disorders

## Abstract

The concept of intersectionality has gradually been introduced to health inequality research, adding depth and breadth to the way inequalities in health are approached. We conducted a scoping review with the purpose to systematically map, describe and analyze the literature about intersectional inequalities in mental health. For eligibility, the study had to analyze and report inequality defined by combinations of socioeconomic position, gender, race/ethnicity, sexual orientation or religion. The mental health outcome had to be measured as self-reported symptoms assessed through validated scales, or disorders assessed through diagnostic interviews. The search strategy was applied in two databases and the screening process yielded 20 studies. The interaction of intersectional positions showed no consistent patterns in mental health across studies, but both synergistic and antagonistic effects were observed. In most studies an absolute measure of inequality was used and few studies analyzed factors potentially explaining the intersectional inequalities. Taken together, the findings of this review highlight the value of assessing intersectional inequalities across population groups for priority setting and action on mental health inequalities.

**Electronic supplementary material:**

The online version of this article (10.1186/s12939-019-1012-4) contains supplementary material, which is available to authorized users.

## Introduction

Health is not equally distributed in the population, which is a concern in public health policy worldwide [1–3]. The distribution of health commonly follows a social gradient where groups advantaged in terms of power, resources and influence display better health than disadvantaged groups. Yet, when comparing health outcomes across combined groups by for example gender, race/ethnicity, socioeconomic position, unexpected patterns may arise, which potentially depend on the interplay between various social positions [4]. This interplay comes into particular focus through the lens of intersectionality.

The concept of intersectionality has gradually been introduced to health inequality research, adding depth and breadth to the way inequalities in health are approached [5, 6]. The term intersectionality was first coined by Kimberlé Crenshaw in 1989, and refers to the interaction and interplay between multiple identities marked differently by dominance and oppression [7]. When considering multiple social positions simultaneously and how their interaction impacts on health outcomes, important differences within groups have been highlighted [4]. A central theme is, for example, that the study of the binary gender categories of “men” and “women” is too broad to provide actionable evidence for health policy, as they comprise subgroups of individuals carrying different life experiences and health needs depending on other characteristics such as ethnicity, income or sexual orientation [8, 9]. A common hypothesis linked to the intersectional approach is that simultaneously experienced disadvantages tend to produce more than additive disadvantage in health [10, 11]. The degree of such effects on health can be studied through quantitative approaches [12, 13], which have been applied in studies of outcomes such as cancer [11], self-rated health [14, 15] and mental health [16]. In all, an intersectional approach may offer a more nuanced description and understanding of what to promote among whom in order to reduce inequalities in health.

Mental ill-health represents a large and growing public health problem globally [17]. Depressive disorders alone are ranked as the third biggest contributor to disability in the world according to the Global Burden of Disease (GBD) study [18] while anxiety ranks as number 9 and schizophrenia number 12 [18]. A large body of research has also identified social inequalities in mental health across multiple dimensions of inequality, such as socioeconomic position, gender, and sexual orientation, among others [19–22]. However, additional dimensions of inequalities may need to be incorporated into the analysis in order to identify complex and potentially unexpected patterns in the distribution of health. For example, one study suggests that complex processes of leveraging of resources shape the inequalities between middle groups in the intersection of economic affluence and gender [16]. Another dynamic is suggested in a different study, which shows that the negative incongruent (middle) group, defined by having a university level education but a manual occupation, were at the highest risk for multiple negative emotional outcomes [23]. In a newly published systematic review about intersectional inequalities across combinations of race/ethnicity and gender, focusing on mental health among adolescents in the United States (US), the included studies were found to be very heterogeneous in terms of study design, analytical approach and in their use of an intersectionality framework [24]. To our knowledge there is however no literature review, either scoping or synthesizing the current research literature, on intersectional inequalities in mental health in the general adult population.

Increased knowledge about mental health across intersecting positions and about which analytical approaches have been applied could provide a stronger empirical foundation for monitoring trends, making policy-decisions and moving the field of inequality research forward. To address this gap we conducted a scoping review with the purpose to systematically map, describe and analyze the literature about intersectional inequalities in mental health.

## Method

The scoping review followed the methodology described by Arksey and O’Malley in 2005 [25] and further refined by Levac in 2010 [26]. They suggest and elaborate on five central methodological steps: 1) Identifying the research question, 2) Identifying relevant studies, 3) Selecting studies, 4) Charting the data, and 5) Collating, summarizing, and reporting results, as well as a sixth optional step: 6) Consultation. Furthermore, we embraced the definition by Colquhoun and colleagues from 2014, which points out that a scoping review “addresses an exploratory research question aimed at mapping key concepts, types of evidence, and gaps in research related to a defined area or field by systematically searching, selecting, and synthesizing existing knowledge”.

### Identifying the research question

Through an iterative process of trial and error, discussions and consultations the scope of the review was formulated in terms of aim, research questions and eligibility criteria. For example, several dimensions of inequality were considered using the PROGRESS-Plus framework [27]. The dimensions finally included were: socioeconomic position (education, income, occupational class, etc.), gender, race or ethnicity, sexual orientation and religion. Age and disability were at first also considered but finally excluded. Disability was excluded mostly due to the difficulties found with respect to the screening process in which studies with mental disability as an outcome had to be discriminated from studies with mental disability as an exposure. We came across other difficulties with age as it is routinely included in analytical models as a covariate and thus difficult to both identify from abstract and interpret. The scope was also limited to high-income settings in order to avoid too much heterogeneity related to for example norms about LGBT-persons, welfare systems and division of labor between women and men.

In addition to the overall aim of mapping, describing and analyzing intersectional inequalities in mental health, four research questions were formulated in order to provide a roadmap for the subsequent stages:

1. Which are the intersectional social positions studied?
2. How has intersectional inequality been operationalized?
3. Which intersectional inequalities in mental health emerge in:
  the individual studies?
  across the synthesized studies?
4. Which explanatory factors have been analysed with respect to intersectional inequalities?

While the aim guided the development of the search strategy and the selection of studies, the research questions were applied to explore the finally included studies.

### Identifying relevant studies

**Fig. 1:**
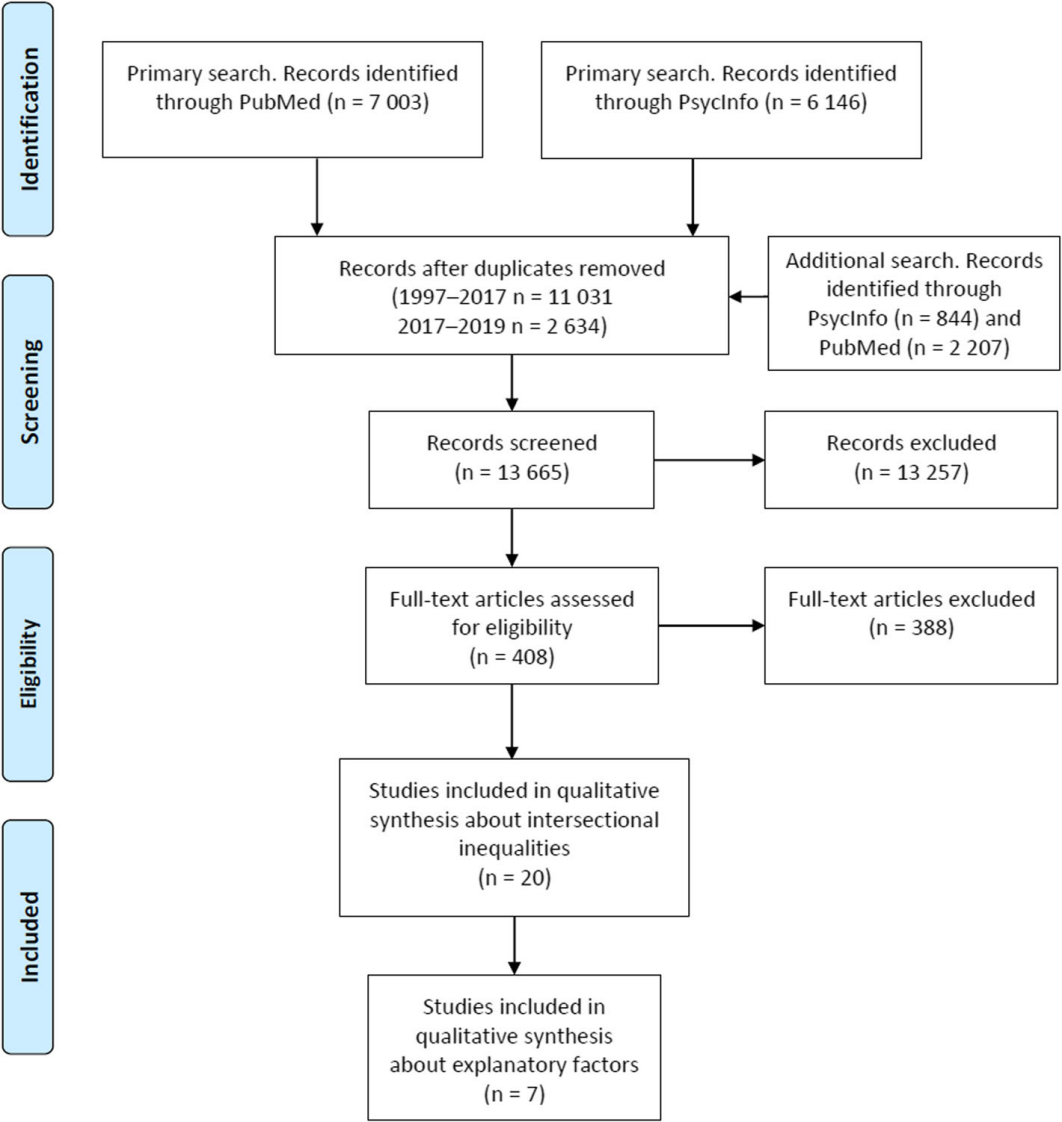
Search process illustrated in a PRISMA Flow diagram

The search strategy was developed together with an information specialist. Due to the breadth of the search, and thus the large number of studies identified, the search was limited to two electronic databases: PsychInfo (American Psycholological Association, APA database) and the National Library of Medicine’s PubMed (including Medline). No language restrictions were applied to the search. Articles in foreign languages provided the title and abstract in English and they could undergo the screening process without translation. The search strategy included two complementing, but still overlapping, searches with three search blocks each. The first search focused on inequalities linked to socioeconomic position, and the second on inequalities linked to well-established dimensions of discrimination: gender, race/ethnicity, religion and sexual orientation. Both searches included a block defining the outcome, i.e. aspects of mental ill-health. The full electronic search strings, which comprised both Mesh terms and free terms, is provided as Additional file 1. The original period applied to the search was 1st January 1997 to 26th January 2017. A second search for papers published between 27th January 2017 and 25th January 2019 was added using the same search terms. The full search process is illustrated in the flow diagram (Fig. 1).

### Screening and selecting studies

Primary research studies from high-income settings, and with a majority of the participants over 18 years, were included. Thus, editorials, letters and reviews were excluded. For eligibility, the study also had to analyze and report inequality defined by intersections of socioeconomic position (education, income, occupational class, etc.), gender, race/ethnicity, sexual orientation or religion. Finally, the mental health outcome could either be self-reported symptoms assessed through validated scales, or mental health disorders assessed through diagnostic interviews.

The search result was imported to the reference management software EndNote. A specific layout and screening procedure described by Bramer et al. in 2017 was used for screening efficiency [28]. Two researchers (AM and NFT) assessed the literature for eligibility independently from each other in two stages: first, title and abstract screening and second, full text screening. An initial meeting was held after screening title and abstracts of a few hundred studies. The purpose of the meeting was to clarify any questions regarding the interpretation of the eligibility criteria in order to ensure inter-rater reliability. A similar meeting took place in the process of screening the studies selected for full text reading. Any disagreement between reviewers in the full text screening process was resolved with a third party (PEG). The result from the second search was screened by NFT and the studies included for full text screening by both NFT and AM.

### Charting the data

As a tool for systematic data extraction, a chart was developed by the authors (AM, PEG and NFT). The data chart initially included columns for the basic characteristics of the studies such as publication year and author. It was further developed to include information on study composition by columns for study context, population size and age, and outcome measure. Finally, columns for data specifically corresponding to the research questions were included in the chart, i.e. the intersections investigated, how intersectionality was operationalized, the direction of any statistically significant intersectional inequality and how it was estimated in terms of absolute and relative measures (mean difference or odds ratio (OR)) as well as potential explanatory factors and their explanatory value. The data was chartered by NFT and cross-checked by the other authors (AM and PEG).

For the extraction of data on intersectional methods and results, we adopted the terms used by Jackson et al. as a terminological framework [29]. According to this, intersectional inequality in health between the two doubly disadvantaged and the doubly privileged position is called the *joint intersectional disparity* and the inequality between the middle groups and the doubly advantaged position as the *referent disparities*. The difference in health between the joint intersectional disparity and the sum of the two referent disparities equates to the *excess intersectional disparity,* which corresponds to the interaction effect between the two intersecting positions. If the excess intersectional inequality was found to be positive, it was labelled as having a synergistic effect, and if it was negative, it was labeled an antagonistic effect. The original approach by Jackson et al. use these terms solely for absolute inequalities (e.g. mean difference), but for the purpose of this review we also apply the terms to relative inequalities (e.g. OR).

### Assessing the risk of bias

We decided to assess and report the risk of bias in the individual studies in a separate chart. Since published and validated risk of bias tools are not designed for assessing studies about inequalities, even less about intersectional inequalities, an assessment guide was developed. Social inequalities in health is a phenomenon that can be constructed as a statement about the population distribution, and there is no evident way to handle confounding vs. mediating factors in relation to exposure and outcome. We therefore focused less on items concerning a causal relationship. The following five domains of quality and risk of bias were critically assessed:

1. Study design

The study is preferably primarily designed to analyze intersectional inequalities (e.g. stated as aim or objective and not included as post hoc analysis).

2) Data collection

The study population is randomly selected and preferably representable of the general population. The collection of data on background characteristics such as income and social class is preferably based on registers, and personal characteristics such as sexual orientation is based on validated questions or questionnaires.

3) Outcome measures

Validated scales or diagnostic tools should be used to measure the outcome.

4) Statistical methods

Preferably statistical methods yielding results corresponding to the intersectional inequalities described in [29] are applied.

5) Reporting of intersectional inequalities

Preferably, all results, not only significant estimates, are reported.

Two authors (NFT and AM) assessed the included studies independently from each other, and a third party (PEG) resolved disagreements, with the exception that the authors did not assess any of their own articles. For each item fulfilled, one point was rewarded, thus the maximum quality ranking per study was five points. The assessment was done to provide a general overview of the quality of the results extracted into this review and not as part of the eligibility process.

### Collating, summarizing, and reporting the results

The charted data was primarily collected and combined according to the intersections identified, for example, the intersection of race/ethnicity and gender. The literature was described according to its basic characteristics, the number of studies covering the specific intersection, and the way intersectional inequalities in health was operationalized according to the terminology of Jackson et al. [29]. For each intersection, we summarized the direction of a statistically significant intersectional inequality, and how it was estimated. The reporting of these summaries as well as data about explanatory factors were structured according to outcome measure and type of intersectional inequality. In this way, data was gradually configured into a descriptive narrative of the literature about intersectional inequalities in mental health.

### Consultation

The review was commissioned, as part of a governmental assignment, by the Public Health Agency of Sweden, which is the national body responsible for monitoring health inequalities in the Swedish population. As such, the progress and results were shared and discussed continuously throughout the work with representatives of the agency. This kind of stakeholder involvement was regarded as important in order to increase the uptake of results, including the intersectional analytical approaches described and discussed in the article which could be used to refine national and regional monitoring. Consultations were done by providing preliminary drafts for feedback, and through dialogue according to pre-specified dates.

## Results

### Study selection

In total, 20 articles were included for analysis (Fig. 1). The most frequent reason for exclusion was that the articles did not include an analysis based on intersectional social positions, i.e. did not study the combination of two or more dimensions of inequality in mental health.

### Study characteristics

The literature identified was published between 2000 and 2019 and included analyses were based on the following combinations: race/ethnicity in combination with gender; socioeconomic position (as indicated by education, income, or occupational class) in combination with gender, race/ethnicity, gender and socioeconomic position; sexual orientation in combination with gender; and different indicators of socioeconomic position in combination with each other. We found no studies about the intersection of religion in combination with another social position.

A summary of the basic characteristics of the included studies is presented in Table 1. The age of the study population ranged from 15 to 84 years and the size from 314 to 83 395 participants. Most of the studies were conducted in the USA (*n* = 13), whereas three in the UK, three in Sweden and one in Canada. The mental health outcome was measured with a clinical interview in one study [30], and with symptom scales in all the other studies (Table 1). The excess intersectional inequality was the most common way of operationalizing intersectional inequality, but also the joint intersectional inequality was estimated in a few studies. Generally, the risk of bias within studies was low. The most common source of bias was that the study was not primarily designed to analyze intersectional inequalities which was the case in four of the included studies [31–34]. The complete final result from the quality assessment is reported in Additional file 2.

**Table 1:** Study characteristics including author, publication year, population, outcome, analytical approach, intersectional inequality and quality rating

Author and publ. Year	Population (sample, age, setting and size)	Outcome measure	Analytical approach	Intersectional inequality (direction of association)	Quality rating
Gender and Race/ethnicity					
Mair C., 2010 [34]	Population sample > 60 yrs., US (*n* = 10 441)	Symptom scale: Center for Epidemiologic Studies Depression Scale (CES-D) (depressive symptoms)	Ordinary least squares regression, absolute measure of inequality	Excess (antagonistic)	4/5
Evans, C. R. and Erickson N., 2019 [31]	Population sample, age wave 1: 15, wave 2: 28 yrs., US (*n* = 15 388)	Symptom scale: CES-D (depressive symptoms)	Linear regression, absolute measure inequality	Excess: Female and Native American (synergistic) Female and Black (n.s) Female and Latina (n.s) Female and Asian/Pacific Islander (n.s)	5/5
Hardeman R., et al., 2015 [32]	Medical students, US (*n* = 3 191)	Symptom scale: Patient-Reported Outcomes Measurement Information System (PROMIS) (depressive symptoms)	Generalized linear regression, relative measure of inequality	Excess (n.s)	5/5
		Symptom scale: PROMIS (anxiety symptoms)		Excess (antagonistic)	5/5
Rosenfield S., 2012 [29]	Data set 1: population sample 15–54 yrs., US (*n* = 5 877)	Diagnostic interview: Composite International Diagnostic Interview (CIDI) (antisocial personality disorder and conduct disorder)	Logistic regression, absolute measure of inequality	Excess (synergistic)	5/5
		Diagnostic interview: CIDI (depression)		Excess (n.s)	5/5
	Data set 2: State sample (New Jersey) US 15,18 and 21 yrs. (*n* = 1 308,)	Symptom scale: Hopkins Symptom Checklist (HSCL)-90R (antisocial problems)		Excess (n.s)	5/5
		Symptom scale: HSCL-90R (depressive symptoms)		Excess (n.s)	5/5
Roxburg S., 2009 [33]	Population sample 18–64 yrs., US (*n* = 24 998)	Symptom scale: Kessler Psychological Distress Scale (K6) (psychological distress)	Ordinary least squares regression, absolute measure of inequality	Excess (n.s)	5/5
Gender and Socioeconomic position					
Green M. J. and Benzeval M., 2011 [34]	Age at baseline: 15, 35 and 55. Follow-up time: 20 yrs. Scottland, UK (*n* = 3846)	Symptom scale: Hospital Anxiety and Depression Scale (HADS) (depressive symptoms)	Logistic regression, absolute measure of inequality	Excess (n.s)	3/5
		Symptom scale: HADS (anxiety symptoms)		Excess (synergistic)	4/5
Green M., et al., 2014 [35]	Age at baseline: 36. Follow-up time: 20 yrs., Skottland, UK (*n* = 999)	Symptom scale: General Health Questionnaire (GHQ) -12 (depressive symptoms)	Structural equation model with latent variables, absolute measure of inequality	Excess (n.s)	4/5
Gibson P. A., et al., 2016 [36]	18–26 yrs. (*n* = 4302), USA	Symptom scale: CES-D (depressive symptoms)	Nested negative binomial regression, absolute measure of inequality	Excess (n.s)	5/5
Ross C. E., and Mirowsky J., 2006 [37]	Population sample, 18–95 yrs., US (n = 2 592)	Symptom scale: CES-D (depressive symptoms)	Ordinary least squares regression, absolute measure of inequality	Excess (synergistic)	4/5
Schieman S., 2002 [38]	Workers 18–55 yrs., Canada (*n* = 994)	Symptom scale: CES-D (depressive symptoms)	Ordinary least squares regression, absolute measure of inequality	Excess (synergistic)	5/5
				Excess (n.s)	
Gustafsson P., et al. 2016 [16]	National sample 18–84 yrs., Sweden (*n* = 25 585)	Symptom scale: GHQ-12 (depressive symptoms)	Analysis of variance (Aim 1) and Blinder-Oaxaca decomposition analysis (Aim 2), absolute measure of inequality	Joint (significant)	5/5
Socioeconomic position and Race/Ethnicity					
Valdez L. A., and Langellier B. A., 2015 [39]	> 18 yrs., US (*n* = 6 070)	Symptom scale:Kessler 6 (psychological distress)	Linear regression, absolute measure of inequality	Excess: household income and ethnicity (n.s)	4/5
				Excess: education and ethnicity (n.s)	
Gender and Race/ethnicity and Socioeconomic position					
Wamala et al., 2009 [40]	National sample 16–84 yrs., Sweden (*n* = 56 889)	Symptom scale:GHQ-12 (depressive symptoms)	Logistic regression, relative measure of inequality	Joint (significant)	5/5
Rosenfield S., 2012 [29]	Data set 1: population sample 15–54 yrs., US (*n* = 5 877)	Diagnostic interview: Composite International Diagnostic Interview (CIDI) (antisocial personality disorder and conduct disorder)	Logistic regression, absolute measure of inequality	Excess (n.s)	5/5
		Diagnostic interview: CIDI (depression)		Excess (n.s)	5/5
	Data set 2: State sample (New Jersey) US15,18 and 21 yrs. (*n* = 1 308,)	Symptom scale: Hopkins Symptom Checklist (HSCL)-90R (antisocial problems)		Excess (n.s)	5/5
		Symptom scale: HSCL-90R (depressive symptoms)		Excess (n.s)	5/5
Sexual orientation and Gender					
Becker M., et al., 2014 [41]	18–28 yrs., US (*n* = 2 451)	Symptom scale: CES-D (depressive symptoms)	Analysis of variance (ANOVA), absolute measure of inequality	Excess (n.s)	5/5
		Symptom scale: SIS (suicidal ideation)		Excess (n.s)	
Li G., et al., 2016 [42]	Mean age 21 yrs., US (*n* = 9 421)	Symptom scale: CES-D (depressive symptoms)	ANOVA, absolute measure of inequality	Excess (n.s)	5/5
Cohen J. M., et al., 2016 [43]	Mean age 18 yrs., US (*n* = 314)	Symptom scale: Generalized Anxiety Disorder Questionnaire (GAD-Q)-9 (anxiety symptoms)	Multivariate analysis of variance (MANOVA), absolute measure of inequality	Excess (n.s)	5/5
		Symptom scale: Posttraumatic Stress Disorder Checklist for DSM-5 (PCL-5) (post traumatic stress symptoms)		Excess (n.s)	
		Social Phobia Diagnostic Questionnaire (SPDQ) (social phobia symptoms)		Excess (n.s)	
		Beck Depression Inventory (BDI) -II (depressive symptoms)		Excess (n.s)	
Strong S. M., et al. 2000 [44]	18–32 yrs., US (*n* = 412)	Symptom scale: BDI (depressive symptoms)	Stepwise multiple regression, absolute measure of inequality	Excess (n.s)	5/5
		Symptom scale: Eating Attitude Test (EAT-26) (eating disorder symptoms)	Chi-square test, absolute measure of inequality	Joint (significant)	
Davids C. M., and Green M. A., 2011 [45]	18–80 yrs., US (*n* = 454)	Symptom scale: Eating Disorder Examination Questionnaire (EDE-Q) (eating disorder symptoms)	Multivariate analysis of covariance (MANCOVA), absolute measure of inequality	Excess (n.s)	4/5
Lundberg J., et al., 2009 [23]	18–70 yrs., Sweden (*n* = 14 854)	Symptom scale: GHQ-12 (common mental disorder symptoms)	Logistic regression, relative measure of inequality	Joint (significant)	5/5
Garratt E., A., et al., 2016 [46]	Parents to children born 2000–01, UK (*n* = 83 395)	Symptom scale: Kessler 6 (common mental disorder symptoms)	Linear fixed-effects panel regression, absolute measure of inequality	Excess (antagonistic)	5/5

### Summary by intersection

#### Race/ethnicity and gender

Five studies, comprising 12 analyses, reported on inequalities in mental health based on the intersection of race/ethnicity and gender [30, 34–37]. The excess intersectional inequality for depression and depressive symptoms was estimated in eight analyses. Four studies used absolute measures of inequality [30, 34–37] and one relative measures [35]. The results showed an antagonistic effects in two of the studies [34, 35] meaning black women had a lower risk for depressive symptoms and symptoms of anxiety than expected given their doubly disadvantaged position. In one study the result showed a synergistic effect for Native American Women [37] . The results from the other nine analyses were non-significant [36, 37].

The excess intersectional inequality was also estimated for two other mental health outcomes (anti-social problems and disorders) [30]. The results showed a synergistic effect for anti-social personality disorder and conduct disorder combined and were non-significant for anti-social problems. The last study, in which the excess intersectional inequality was estimated for psychological distress, reported non-significant results [36].

#### Socioeconomic position and gender

In total, seven studies comprising 12 analyses reported inequalities in mental health based on the intersection of socioeconomic position and gender [16, 30–33, 38, 39]. Intersectional inequality was estimated as the excess intersectional inequality in all but one study, which estimated the joint intersectional inequality [16]. In all studies, absolute measures of inequality were used. Four of the analyses showed synergistic effects among women with low social position (indicated by either occupational class or education) for symptoms of anxiety [32], anti-social personality disorder and conduct disorder combined [30] and depressive symptoms [38, 39]. The other analyses found no significant effects.

### Explanatory factors

Four of the studies explored potential explanatory factors of the intersectional inequalities in mental health [16, 33, 38, 39]. Green et al. looked into whether insomnia explained the excess intersectional inequality for depressive symptoms and found that it did not [33]. Gustafsson et al. looked into to what degree a range of material and psychosocial factors contributed to the inequality in depressive symptoms between intersectional middle groups (referent inequalities) [16]. The results showed that the factors included in the analysis contributed between 33 and 75% to the referent inequalities. The psychosocial factors (violence and degrading treatment) contributed to 80% of the inequality between middle-income women and middle-income men while the material factors (cash margin, diff. Making ends meet, residential ownership) contributed with an equal proportion to the inequality between middle and low-income men. A third (33.5%) of the inequality between high- and middle-income women was explained by the factors included in the analysis. Ross and Mirowsky looked into whether marriage, income and workplace related factors contributed to the excess intersectional disparity in depressive symptoms [38]. The results showed that the included factors contributed with 50% to the excess intersectional inequality and was no longer significant when these factors had been taken into account. Shieman looked into whether sense of control in one’s life and self-esteem contributed to the excess intersectional inequality in depressive symptoms [39]. The results showed (also in this analysis) that the included factors contributed with 50% to the excess intersectional inequality, and the inequality was no longer significant when these factors had been taken into account.

#### Race/ethnicity and socioeconomic position

One study comprising two analyses report on inequalities in mental health based on the intersection of race/ethnicity and socioeconomic position [40]. An absolute measure of inequality was used. The excess intersectional inequality was estimated for psychological distress but did not show statistically significant results.

#### Race/ethnicity, gender and socioeconomic position

Two studies reported on inequalities in mental health based on the three-way intersection of race/ethnicity, gender and socioeconomic position [30, 41]. In one study, the intersectional inequality was estimated as the absolute excess intersectional inequality. It showed a non-significant result for all four outcome measures (depressive symptoms and depression, anti-social problems and anti-social disorders) [30]. The other study estimated the joint intersectional inequality and used a relative measure of inequality. It showed a 2.73 times higher odds of depressive symptoms among low-income women born outside Sweden than among high-income men born in Sweden [41].

### Explanatory factors

One of the studies explored potential explanatory factors of the intersectional inequalities in mental health. The study looked into whether self-salience contributed to the referent inequalities in depression, depressive symptoms, antisocial personality disorder, conduct disorder, and anti-social problems [30]. Self-salience contributed with two thirds to the inequality in depression between highly educated black and white women, and the observed inequality was no longer significant after adjusting for it. The analyses including the other outcomes were non-significant even in the unadjusted analyses.

#### Sexual orientation and gender

Five studies comprising ten analyses reported on inequalities in mental health based on the intersection of sexual orientation and gender [42–46]. All studies used absolute measures of inequality and estimated the intersectional inequality as the excess intersectional inequality. The results were not statistically significant in any of the studies. However, Strong et al. also estimated the joint intersectional inequality in symptoms of eating disorder, which showed a statistically significant difference [45].

#### Different socioeconomic indicators

Two studies reported on inequalities in mental health based the intersection of different socioeconomic indicators, one using an absolute measures of inequality [47] and one using a relative [23]. As different socioeconomic indicators, absolute income and income rank were used in one study [47] and education and occupational class were used in the other [23]. In the study by Lundberg et al. the intersectional inequality was estimated as the largest difference identified between the four groups which were compared [23]. The results showed that individuals with high education and a manual occupation had the highest prevalence of depressive symptoms while individuals with low education and non-manual occupation had the lowest prevalence. Furthermore, the individuals with low education and manual occupation had a lower prevalence of depressive symptoms than individuals with high education and non-manual occupation. In the other study, by Garratt, the intersectional inequality was estimated as the excess intersectional inequality for psychological distress, which was significant [47]. The result showed an antagonistic effect, meaning that doubly disadvantaged individuals reported better mental health outcomes than expected considering their low income and low income rank separately.

### Explanatory factors

One of the studies explored potential explanatory factors of the intersectional inequalities in mental health. Lundberg et al. looked into whether gender, age, economic hardship and long lasting illness contributed to the inequality between individuals with high education and low income and individuals with low education and high income [23]. The included factors contributed with approximately one sixth of the inequality, which was attenuated below significance after adjustment for all the factors.

## Discussion

This scoping review demonstrates that research about intersectional inequalities in mental health across groups is still limited in volume and not yet methodologically standardized. It was therefore not possible to conclude any general mental health patterns across any specific intersectional positions, which could, for example, confirm or reject the hypothesis of multiple jeopardy. Nevertheless, there are relevant findings for future research, practice and policy, which are discussed in the following sections.

### Intersections

When the results from the 20 eligible studies were divided and grouped according to intersectional positions and mental health outcome, the basis for analysis became small, and for some intersections no studies were found. Bauer raises the question of whether all intersectional identities are of equal or sufficient value to study [5]. There is obviously no given answer to this question, at least not given the scant literature in the field and as long as there are intersections that are scarcely studied. Nevertheless, by applying different normative perspectives different arguments to the question are emphasized. For example, the size of the intersectional group, in combination with the magnitude of ill health, has been raised as an important argument to consider [48]. Another argument is expressed in the United Nation Agenda 2030 to achieve the Sustainable Development Goals which emphasizes the importance of reaching those furthest behind [49]. Moreover, the possibility to decrease the inequalities by societal effort and reasonable resources have been raised as additional important aspects to consider [50]. Besides different ethical premises underlying such propositions, the relevance of studying a certain intersection of social positions is of course dependent on context. One example is the classification of race and ethnicity, which is used in North America but controversial therefore of limited value for practice and policy in Europe. Nevertheless, this review shows that there are potentially relevant intersections left to be explored, such as the intersection of sexual orientation and socioeconomic position as well as intersections including religion.

### Analytical approaches

Depending on the analytical approach used to estimate intersectional inequalities, different aspects of inequality may be explored and highlighted. We found that the excess intersectional inequality, corresponding to the interaction term in additive or multiplicative regression models, is the most frequent analytical approach in the field of mental health. In contrast, the joint intersectional inequality, corresponding to the health inequality between the doubly advantaged and doubly disadvantaged group, is rarely estimated. Further, even though the included studies are based on some kind of regression-based interaction analysis, they differ regarding specific analytical approach and what aspect of intersectional inequalities they capture. From a policy point of view, the underlying purpose of and need for studying inequalities is of primary importance to articulate before the analytical approach is chosen.

The estimation of absolute and relative inequalities is one particular issue highlighted in this review, in which absolute measures were identified in 37 of 40 analyses. Considering that absolute and relative measures capture different facets of inequalities and do not necessarily correspond to each other, it has been emphasized that studies on health inequalities routinely should measure both [51]. Whereas absolute inequalities have been argued to be of greater public health relevance, as it is sensitive to the level of health in the population, relative inequalities are considered more appropriate when evaluating the strength of association and comparisons between different populations [51]. These notions apply, and should also be taken into consideration, when studying intersectional inequalities. However, in the reviewed studies the choice of absolute or relative inequality measure appears to have been guided more by the scale level of the outcome and the standard type of regression model applied to that situation, i.e. additive for continuous outcomes and multiplicative for binary outcomes. Research on intersectional inequalities would benefit from taking these fundamental issues about health inequality measurement into account, to produce the most policy-relevant knowledge.

Furthermore, the issue of measuring intersectional inequalities is under ongoing debate and development. In addition to the interaction-based method proposed by Jackson et al. [29], a number of more advanced statistical methods for measuring magnitude have recently been proposed and applied on, for example, those based on discriminatory accuracy [52] and multilevel analysis [37, 53]. In addition, novel approaches are emerging to explain these inequalities, which in this review is exemplified by a study using decomposition analysis [16]. In summary, the way we measure and seek to explain have meaningful conceptual and empirical value, and in the end policy implications, and should be heeded in future research on intersectional inequalities in health.

### Intersectional synergism or antagonism

When it comes to the results describing excess intersectional inequalities, we found that most studies demonstrated a synergistic effect, but with a few studies concluded an antagonistic effect. When synergistic, it supports the multiple jeopardy hypothesis, i.e., the experience of multiple disadvantage has more than an additive impact on health [10, 13]. On the other hand, when antagonistic, the multiple disadvantage has less than an additive impact on health. In this review, two studies were found in which black women had a lower risk for depressive symptoms and symptoms of anxiety than expected given their doubly disadvantaged position. These results can possibly be related to the so called “Black-White Mental Health Paradox”, which highlights that blacks have similar or lower rates of mental disorders than whites even though they are more exposed to stress and experience greater economic disadvantage [54, 55]. The results supports the conclusion drawn in a review about this topic which states that the paradox is mainly due to the low rates of internalizing problems among (black) women [56]. Although the results of the present review does not shed light on the underpinnings of the patterns, the paradox can possibly be understood from the combination of gender relations and stress, coping, personal relationships and resources/vulnerabilities [56].

No general patterns identifying a particularly disadvantaged group could be identified across the studies in this review. Since the literature stretched over different contexts, such generalizations may nonetheless be of limited policy-relevance from a global or national perspective. The lesson learnt is rather that intersectional inequalities in mental health are not predictable simply by the particular social positions combined, and that the health impact across intersectional positions therefore need to be empirically assessed in different settings. From a policy-making point of view, specific and context-relevant knowledge about such inequalities across groups is of central value for priority setting and action. However, since such knowledge based on quantitative intersectionality research may involve certain limitations such as small sample sizes (e.g. of certain ethnic or sexual minority groups), it could benefit from integration with knowledge from qualitative studies. Furthermore, when its implications for policy are difficult to interpret (i.e when doubly disadvantaged groups in the social hierarchy don’t show the worst health outcomes), a stronger normative guidance could emerge when incorporated into ethical arguments regarding a fair distribution of population health.

### Explanatory factors

We found few studies that analyzed factors potentially explaining the intersectional inequalities in mental health and those who did differed in character and scope. Some studies simultaneously examined explanatory factors of both material and social content [16], whereas other studies examined a more limited set, such as factors related to working life [38], insomnia [33] sense of control and self-esteem [39], self-salience [30], or predominantly demographic variables [23, 36]. It is generally difficult to draw conclusions from analyzes with only a few factors included, as it is unclear to what extent these factors explain the inequalities themselves or through unconsidered factors.

In several studies, explanations were examined for intersectional inequalities that themselves were not statistically significant, which is not particularly informative. Furthermore, the studies were often poorly designed to facilitate the explanation of inequalities. Only one study [16] applied decomposition analysis, which is adapted for explanatory purpose, whereas the other studies used linear or logistic regression analyzes, which do not directly distinguish the unique contribution from an individual factor to the health inequality per se, in a model with many other factors. Knowledge on the mechanisms behind intersectional inequalities is the very foundation for being able to reduce them, and this is why we suggest future research on this topic.

### Limitations

We deemed that a scoping review with the intention to describe and analyze intersectional mental health inequalities, but not to aggregate specific outcomes, was an ideal choice. Nevertheless, the review process was associated with several limitations. One challenge was related to the identification of literature. As few studies explicitly used intersectionality as a theoretical point of departure, likely explained by the novelty of the concept within quantitative public health, the concept was rarely mentioned in the abstract or even in the full text paper. This made it challenging to identify the relevant literature, i.e. finding the articles that are informative for our purpose but explicitly lack an intersectional conceptual framework. Balancing the risk of excluding relevant articles and including relevant ones was therefore essential when developing the search strategy.

Even though only two databases were used, the final searches generated a large number of records. Adding databases to our search strategy would have generated even more records that would have posed an even larger challenge to the manual screening process. Instead of applying filters to the searches or adding search terms to limit the number of studies to screen, extra amount of time was allocated to the screening process. This is according to the recommendation of the Preferred Reporting Items for Systematic reviews and Meta-Analyses (PRISMA)-Equity extension guideline, which states that the risk of missing relevant articles is too high otherwise [57].

It is also important to point out that we strictly applied the eligibility criteria of validated scales or diagnostic interviews regarding the outcome of mental health. It is possible that the included literature would have been richer in volume if this has been applied less strict. However, this limitation in scope was considered to overweight the potential disadvantage of results being more difficult to interpret.

Finally, it is important to point out that the scope of this review does not cover all mental health problems. For example, substance use and addiction were not included in this review, but make up a significant part of the global mental health burden and are more frequent among men [18]. For future research we therefore suggest a review focusing on externalizing problems such as aggression, substance use and addiction disorders which are more prevalent among men.

## Conclusion

The literature about intersectional inequalities in mental health covered multiple intersecting social positions, albeit not all possible combinations. When it comes to analytical approaches, most studies estimated absolute excess intersectional inequalities. Both synergistic and antagonistic effects of intersectional positions were observed, which suggests interacting power dynamics that otherwise would have been concealed if approached in a disentangled manner, as is standard in health inequality research. However, no general patterns across studies were found regarding any particularly disadvantaged position or intersectional inequality. Few studies analyzed factors potentially explaining the intersectional inequalities in mental health, and those who did differed in character and comprehensiveness. Taken together, the findings of this review highlight the value of assessing intersectional inequalities across population groups for priority setting and action on mental health inequalities. This review also shows that there are intersections left to be explored, and for future research we particularly suggests that the underlying purpose of and need for studying inequalities is articulated before the analytical approach is chosen, that both absolute and relative measurements are used, and that quantitative evidence is combined with qualitative to a larger extent.

## Additional files

Additional file 1: Search strings. Full search strings as applied in PubMed and PsycInfo. (PDF 146 kb)

Additional file 2: Quality Appraisal checklist. Items for quality appraisal and final results. (DOCX 29 kb)

## Data Availability

All data generated or analyzed during this study are included in this published article and its supplementary information files.

## Abbreviations

ANOVA: Analysis of variance
BDI: Beck Depression Inventory
CES-D: Center for Epidemiologic Studies Depression Scale
CIDI: Composite International Diagnostic Interview
EAT: Eating Attitude Test
EDE-Q: Eating Disorder Examination Questionnaire
GAD: Generalized Anxiety Disorder
GBD: Global Burden of Disease
GHQ: General Health Questionnaire
HADS: Hospital Anxiety and Depression Scale
HSCL: Hopkins Symptom Checklist
K6: Kessler Psychological Distress Scale
MANCOVA: Multivariate analysis of covariance
MANOVA: Multivariate analysis of variance
OR: Odds ratio
PCL-5: Posttraumatic Stress Disorder Checklist for DSM-5
PRISMA: Preferred Reporting Items for Systematic Reviews and Meta-Analyses
PROMIS: Patient-Reported Outcomes Measurement Information System
SPDQ: Social Phobia Diagnostic Questionnaire
UK: United Kingdom
US: United States
