# Supplementary material for: Languishing in the crossroad? A scoping review of intersectional inequalities in mental health
Source: Int J Equity Health. 2019 Jul 24;18:115. doi: 10.1186/s12939-019-1012-4 (PMC6657170; doi:10.1186/s12939-019-1012-4)
Supplement: Supplementary file 1 — Search strings. Full search strings as applied in PubMed and PsycInfo. (PDF 146 kb) [file 12939_2019_1012_MOESM1_ESM.pdf]

Supplementary file: search strings

PubMed 2017-01-26

**#1 Social position**	
"Family Characteristics"[Mesh] OR "Ethnic Groups"[Mesh] OR "Population Density"[Mesh] OR "Population Groups"[Mesh] OR "Residence Characteristics"[Mesh] OR "Population"[Mesh] OR "Sociological Factors/epidemiology"[Mesh] OR "Sociological Factors/ethnology"[Mesh] OR "Sociological Factors/prevention and control"[Mesh] OR "Sociological Factors/psychology"[Mesh] OR "Sociological Factors/statistics and numerical data"[Mesh] OR “Place of residence”[TIAB] OR race [TIAB] OR ethnicity[TIAB] OR socioeconomic[TIAB] OR occupation[TIAB] OR income[TIAB] OR unemployed[TIAB] OR unemployment[TIAB] OR religion[TIAB] OR education[TIAB] OR “educational status”[TIAB]OR gender[TIAB] OR “Social capital”[TIAB] OR "vulnerable groups"[TIAB] OR marginalized[TIAB] OR "social status"[TIAB]	
AND	
"Social Determinants of Health"[Mesh] OR "Health Status Disparities"[Mesh] OR Inequity[TIAB] OR Inequalities[TIAB] OR inequity[TIAB] OR inequities[TIAB] OR equity[TIAB] OR disparity[TIAB] OR disparities[TIAB] OR intersectional[TIAB] OR intersectionality[TIAB] OR intersectionally[TIAB] OR "double burden"[TIAB] OR "double risk"[TIAB] OR disadvantage[TIAB] OR disadvantaged[TIAB] OR "social gradient"[TIAB]	
AND	
("Mental Health/epidemiology"[Mesh] OR "Mental Health/ethnology"[Mesh] OR "Mental Health/prevention and control"[Mesh] OR "Mental Health/statistics and numerical data"[Mesh] OR "Depression/epidemiology"[Mesh] OR "Depression/ethnology"[Mesh] OR "Depression/prevention and control"[Mesh] OR "Depression/psychology"[Mesh] OR "Depression/statistics and numerical data"[Mesh] OR "Suicide/epidemiology"[Mesh] OR "Suicide/ethnology"[Mesh] OR "Suicide/prevention and control"[Mesh] OR "Suicide/psychology"[Mesh] OR "Suicide/statistics and numerical data"[Mesh] OR "Anxiety Disorders/epidemiology"[Mesh] OR "Anxiety Disorders/ethnology"[Mesh] OR "Anxiety Disorders/prevention and control"[Mesh] OR "Anxiety Disorders/psychology"[Mesh]	

OR “Anxiety Disorders/statistics and numerical data”[Mesh] OR "Bipolar and Related Disorders/epidemiology"[Mesh] OR "Bipolar and Related Disorders/ethnology"[Mesh] OR "Bipolar and Related Disorders/prevention and control"[Mesh] OR "Bipolar and Related Disorders/psychology"[Mesh] OR "Bipolar and Related Disorders/statistics and numerical data"[Mesh] OR "Dissociative Disorders/epidemiology"[Mesh] OR "Dissociative Disorders/ethnology"[Mesh] OR "Dissociative Disorders/prevention and control"[Mesh] OR "Dissociative Disorders/psychology"[Mesh] OR "Dissociative Disorders/statistics and numerical data"[Mesh] OR "Feeding and Eating Disorders/epidemiology"[Mesh] OR "Feeding and Eating Disorders/ethnology"[Mesh] OR "Feeding and Eating Disorders/prevention and control"[Mesh] OR "Feeding and Eating Disorders/psychology"[Mesh] OR "Feeding and Eating Disorders/statistics and numerical data"[Mesh] OR "Mood Disorders/epidemiology"[Mesh] OR "Mood Disorders/ethnology"[Mesh] OR "Mood Disorders/prevention and control"[Mesh] OR "Mood Disorders/psychology"[Mesh] OR "Mood Disorders/statistics and numerical data"[Mesh] OR "Neurocognitive Disorders/epidemiology"[Mesh] OR "Neurocognitive Disorders/ethnology"[Mesh] OR "Neurocognitive Disorders/prevention and control"[Mesh] OR "Neurocognitive Disorders/psychology"[Mesh] OR "Neurocognitive Disorders/statistics and numerical data"[Mesh] OR "Neurotic Disorders/epidemiology"[Mesh] OR "Neurotic Disorders/ethnology"[Mesh] OR "Neurotic Disorders/prevention and control"[Mesh] OR "Neurotic Disorders/psychology"[Mesh] OR "Neurotic Disorders/statistics and numerical data"[Mesh] OR "Personality Disorders/epidemiology"[Mesh] OR "Personality Disorders/ethnology"[Mesh] OR "Personality Disorders/prevention and control"[Mesh] OR "Personality Disorders/psychology"[Mesh] OR "Personality Disorders/statistics and numerical data"[Mesh] OR "Schizophrenia Spectrum and Other Psychotic Disorders/epidemiology"[Mesh] OR "Schizophrenia Spectrum and Other Psychotic Disorders/ethnology"[Mesh] OR "Schizophrenia Spectrum and Other Psychotic Disorders/prevention and control"[Mesh] OR "Schizophrenia Spectrum and Other Psychotic Disorders/psychology"[Mesh] OR "Schizophrenia Spectrum and Other Psychotic Disorders/statistics and numerical data"[Mesh] OR "Somatoform Disorders/epidemiology"[Mesh] OR "Somatoform Disorders/ethnology"[Mesh] OR "Somatoform Disorders/prevention and control"[Mesh] OR "Somatoform Disorders/psychology"[Mesh] OR "Somatoform	

Disorders/statistics and numerical data"[Mesh] OR "Mental health"[TIAB] OR “mental health”[ot] OR depression[TIAB] OR anxiety[TIAB] OR bipolar[TIAB] OR schizophrenia[TIAB] OR “personality disorders”[TIAB] OR “sleep problems”[TIAB] OR wellbeing[TIAB] OR "well-being"[TIAB] OR "mental disorder"[TIAB] OR "health complaint"[TIAB] OR suicide[TIAB] OR suicidal[TIAB] OR anorexia[TIAB] OR bulimia[TIAB]	
**Total records**	5414
**#2 Discrimination**	
“Minority Groups"[Mesh] OR "Sexual Minorities"[Mesh] OR "sexual orientation"[TIAB] OR “sexual minority”[TIAB] OR “sexual minorities”[TIAB] OR “sexual orientation”[TIAB] OR homosexual[TIAB] OR homosexuals[TIAB] OR homosexuals'[TIAB] OR gay[TIAB] OR lesbian[TIAB] OR lesbians[TIAB] OR lesbians'[TIAB] OR LGBT[TIAB] OR LGBQ[TIAB] OR transgender[TIAB] OR transgenders[TIAB] OR bisexual[TIAB] OR bisexuals[TIAB] OR bisexuals’[TIAB]	
AND	
("Family Characteristics"[Mesh] OR "Ethnic Groups"[Mesh] OR "Population Density"[Mesh] OR "Population Groups"[Mesh] OR "Residence Characteristics"[Mesh] OR "Population"[Mesh] OR "Social Determinants of Health"[Mesh] OR "Health Status Disparities"[Mesh] OR "Sociological Factors/epidemiology"[Mesh] OR "Sociological Factors/ethnology"[Mesh] OR "Sociological Factors/prevention and control"[Mesh] OR "Sociological Factors/psychology"[Mesh] OR "Sociological Factors/statistics and numerical data"[Mesh] OR “Place of residence”[TIAB] OR Inequity[TIAB] OR Inequalities[TIAB] OR inequity[TIAB] OR inequities[TIAB] OR equity[TIAB] OR disparity[TIAB] OR disparities[TIAB] OR intersectional[TIAB] OR intersectionalities[TIAB] OR intersectionality[TIAB] OR intersectionality's[TIAB] OR intersectionally[TIAB] OR "double burden"[TIAB] OR "double risk"[TIAB] OR disadvantage[TIAB] OR disadvantaged[TIAB] OR "vulnerable groups"[TIAB] OR marginalized[TIAB] OR "social gradient"[TIAB] OR race [TIAB] OR ethnicity[TIAB] OR socioeconomic[TIAB] OR occupation[TIAB] OR income[TIAB] OR unemployed[TIAB] OR unemployment[TIAB] OR religion[TIAB] OR education[TIAB] OR “educational status”[TIAB]OR gender[TIAB] OR “Social	

capital"[TIAB] OR "vulnerable groups"[TIAB] OR marginalized[TIAB] OR "social status"[TIAB]	
AND	
("Mental Health/epidemiology"[Mesh] OR "Mental Health/ethnology"[Mesh] OR "Mental Health/prevention and control"[Mesh] OR "Mental Health/statistics and numerical data"[Mesh] OR "Depression/epidemiology"[Mesh] OR "Depression/ethnology"[Mesh] OR "Depression/prevention and control"[Mesh] OR "Depression/psychology"[Mesh] OR "Depression/statistics and numerical data"[Mesh] OR "Suicide/epidemiology"[Mesh] OR "Suicide/ethnology"[Mesh] OR "Suicide/prevention and control"[Mesh] OR "Suicide/psychology"[Mesh] OR "Suicide/statistics and numerical data"[Mesh] OR "Anxiety Disorders/epidemiology"[Mesh] OR "Anxiety Disorders/ethnology"[Mesh] OR "Anxiety Disorders/prevention and control"[Mesh] OR "Anxiety Disorders/psychology"[Mesh] OR "Anxiety Disorders/statistics and numerical data"[Mesh] OR "Bipolar and Related Disorders/epidemiology"[Mesh] OR "Bipolar and Related Disorders/ethnology"[Mesh] OR "Bipolar and Related Disorders/prevention and control"[Mesh] OR "Bipolar and Related Disorders/psychology"[Mesh] OR "Bipolar and Related Disorders/statistics and numerical data"[Mesh] OR "Dissociative Disorders/epidemiology"[Mesh] OR "Dissociative Disorders/ethnology"[Mesh] OR "Dissociative Disorders/prevention and control"[Mesh] OR "Dissociative Disorders/psychology"[Mesh] OR "Dissociative Disorders/statistics and numerical data"[Mesh] OR "Feeding and Eating Disorders/epidemiology"[Mesh] OR "Feeding and Eating Disorders/ethnology"[Mesh] OR "Feeding and Eating Disorders/prevention and control"[Mesh] OR "Feeding and Eating Disorders/psychology"[Mesh] OR "Feeding and Eating Disorders/statistics and numerical data"[Mesh] OR "Mood Disorders/epidemiology"[Mesh] OR "Mood Disorders/ethnology"[Mesh] OR "Mood Disorders/prevention and control"[Mesh] OR "Mood Disorders/psychology"[Mesh] OR "Mood Disorders/statistics and numerical data"[Mesh] OR "Neurocognitive Disorders/epidemiology"[Mesh] OR "Neurocognitive Disorders/ethnology"[Mesh] OR "Neurocognitive Disorders/prevention and control"[Mesh] OR "Neurocognitive Disorders/psychology"[Mesh] OR "Neurocognitive Disorders/statistics and numerical data"[Mesh] OR "Neurotic Disorders/epidemiology"[Mesh] OR "Neurotic	

Disorders/ethnology"[Mesh] OR "Neurotic Disorders/prevention and control"[Mesh] OR "Neurotic Disorders/psychology"[Mesh] OR "Neurotic Disorders/statistics and numerical data"[Mesh] OR "Personality Disorders/epidemiology"[Mesh] OR "Personality Disorders/ethnology"[Mesh] OR "Personality Disorders/prevention and control"[Mesh] OR "Personality Disorders/psychology"[Mesh] OR "Personality Disorders/statistics and numerical data"[Mesh] OR "Schizophrenia Spectrum and Other Psychotic Disorders/epidemiology"[Mesh] OR "Schizophrenia Spectrum and Other Psychotic Disorders/ethnology"[Mesh] OR "Schizophrenia Spectrum and Other Psychotic Disorders/prevention and control"[Mesh] OR "Schizophrenia Spectrum and Other Psychotic Disorders/psychology"[Mesh] OR "Schizophrenia Spectrum and Other Psychotic Disorders/statistics and numerical data"[Mesh] OR "Somatoform Disorders/epidemiology"[Mesh] OR "Somatoform Disorders/ethnology"[Mesh] OR "Somatoform Disorders/prevention and control"[Mesh] OR "Somatoform Disorders/psychology"[Mesh] OR "Somatoform Disorders/statistics and numerical data"[Mesh] OR "Mental health"[TIAB] OR “Mental health”[ot] OR depression[TIAB] OR anxiety[TIAB] OR bipolar[TIAB] OR schizophrenia[TIAB] OR “personality disorders”[TIAB] OR “sleep problems”[TIAB] OR wellbeing[TIAB] OR "well-being"[TIAB] OR "mental disorder"[TIAB] OR "health complaint"[TIAB] OR suicide[TIAB] OR suicidal[TIAB] OR anorexia[TIAB] OR bulimia[TIAB]	
**Total records**	2439
**#1 AND #2 AND ("1997/01/01"[PDAT] : "2017/01/26"[PDAT])**	**7473**

PsycINFO 2017-01-26

**#1 Social position**	
SU.EXACT("Suicide") OR SU.EXACT("Attempted Suicide") OR SU.EXACT("Self-Destructive Behavior") OR SU.EXACT("Anxiety") OR SU.EXACT("Anxiety Disorders") OR SU.EXACT("Social Anxiety") OR SU.EXACT.EXPLODE("Depression (Emotion)") OR SU.EXACT.EXPLODE("Major Depression") OR SU.EXACT("Neurosis") OR	

SU.EXACT("Dementia") OR SU.EXACT("Eating Disorders") OR SU.EXACT("Anxiety Disorders") OR SU.EXACT("Mental Health") OR SU.EXACT("Schizoaffective Disorder") OR SU.EXACT("Hysteria") OR SU.EXACT("Impulse Control Disorders") OR SU.EXACT("Personality Disorders") OR SU.EXACT("Mental Disorders") OR SU.EXACT("Psychosis") OR SU.EXACT("Mental Disorders due to General Medical Conditions") OR SU.EXACT("Affective Disorders") OR SU.EXACT("Chronic Mental Illness") OR if(mental Health OR mental disorder) OR ti,ab("mental health OR "depression OR anxiety OR bipolar OR schizophrenia OR "personality disorders" OR "sleep problems" OR wellbeing OR "well-being" OR "mental disorder" OR "health complaint" OR suicide OR suicidal OR anorexia OR bulimia)	
AND	
SU.EXACT("Homeless") OR SU.EXACT("Poverty") OR SU.EXACT("Race and Ethnic Discrimination") OR SU.EXACT("Gender Equality") OR SU.EXACT("Homeless Mentally Ill") OR SU.EXACT("Social Discrimination") OR SU.EXACT("Employment Discrimination") OR SU.EXACT("Unemployment") OR SU.EXACT("Gender Gap") OR SU.EXACT("Social Issues")OR SU.EXACT.EXPLODE("Socioeconomic Status") OR SU.EXACT("Social Capital") OR SU.EXACT.EXPLODE("Status") OR SU.EXACT.EXPLODE("Income Level") OR SU.EXACT("Social Deprivation") OR SU.EXACT("Social Mobility") OR SU.EXACT.EXPLODE("Social Class") OR SU.EXACT("Equal Education") OR SU.EXACT("Acculturation") OR SU.EXACT("Cross Cultural Differences") OR SU.EXACT("Cultural Deprivation") OR SU.EXACT("Multiracial") OR SU.EXACT("Ethnic Identity") OR SU.EXACT("Culture Change") OR MJSUB.EXACT("Sociocultural Factors") OR MJSUB.EXACT("Social Integration") OR Ti,ab(race OR ethnicity OR socioeconomic OR occupation OR income OR unemployed OR unemployment OR religion OR education OR "educational status" OR gender OR "Social capital" OR "vulnerable groups" OR marginalized OR "social status")	
AND	
SU.EXACT("Human Rights") OR SU.EXACT("Social Equality") OR SU.EXACT("Sex Discrimination") OR MJSUB.EXACT("Social Justice") OR Ti,ab(Inequity OR Inequalities OR inequity OR inequities OR equity OR disparity OR disparities OR intersectional OR intersectionality OR intersectionally OR "double burden" OR "double risk" OR disadvantage OR disadvantaged OR "social gradient")	

Filter: Publication year (1997-2017), scholarly journals	
**Total records**	4043
**#2 Discrimination**	
SU.EXACT(“Suicide”) OR SU.EXACT(“Attempted Suicide”) OR SU.EXACT(“Self-Destructive Behavior”) OR SU.EXACT(“Anxiety”) OR SU.EXACT(“Anxiety Disorders”) OR SU.EXACT(“Social Anxiety”) OR SU.EXACT.EXPLODE(“Depression (Emotion)”) OR SU.EXACT.EXPLODE(“Major Depression”) OR SU.EXACT(“Neurosis”) OR SU.EXACT(“Dementia”) OR SU.EXACT(“Eating Disorders”) OR SU.EXACT(“Anxiety Disorders”) OR SU.EXACT(“Mental Health”) OR SU.EXACT(“Schizoaffective Disorder”) OR SU.EXACT(“Hysteria”) OR SU.EXACT(“Impulse Control Disorders”) OR SU.EXACT(“Personality Disorders”) OR SU.EXACT(“Mental Disorders”) OR SU.EXACT(“Psychosis”) OR SU.EXACT(“Mental Disorders due to General Medical Conditions”) OR SU.EXACT(“Affective Disorders”) OR SU.EXACT(“Chronic Mental Illness”) OR if(mental Health OR mental disorder) OR ti,ab(“mental health OR ”depression OR anxiety OR bipolar OR schizophrenia OR “personality disorders” OR “sleep problems” OR wellbeing OR “well-being” OR “mental disorder” OR “health complaint” OR suicide OR suicidal OR anorexia OR bulimia)	
AND	
(SU.EXACT("Homeless") OR SU.EXACT("Poverty") OR SU.EXACT("Race and Ethnic Discrimination") OR SU.EXACT("Gender Equality") OR SU.EXACT("Homeless Mentally Ill") OR SU.EXACT("Social Discrimination") OR SU.EXACT("Employment Discrimination") OR SU.EXACT("Unemployment") OR SU.EXACT("Gender Gap") OR SU.EXACT("Social Issues") OR SU.EXACT.EXPLODE("Socioeconomic Status") OR SU.EXACT("Social Capital") OR SU.EXACT.EXPLODE("Status") OR SU.EXACT.EXPLODE("Income Level") OR SU.EXACT("Social Deprivation") OR SU.EXACT("Social Mobility") OR SU.EXACT.EXPLODE("Social Class") OR SU.EXACT("Equal Education") OR SU.EXACT("Acculturation") OR SU.EXACT("Cross Cultural Differences") OR SU.EXACT("Cultural Deprivation") OR SU.EXACT("Multiracial") OR SU.EXACT("Ethnic Identity") OR SU.EXACT("Culture Change") OR MJSUB.EXACT("Sociocultural Factors") OR MJSUB.EXACT("Social Integration") OR Ti,ab(race OR ethnicity OR socioeconomic OR occupation OR income OR unemployed OR unemployment OR religion OR education OR "educational status" OR gender OR "Social capital" OR "vulnerable groups" OR marginalized OR "social status"))	

AND	
(SU.EXACT("Minority Groups") OR SU.EXACT("Lesbianism") OR SU.EXACT("Homosexuality") OR SU.EXACT("Heterosexuality") OR SU.EXACT("Male Homosexuality") OR SU.EXACT("Bisexuality") OR MJSUB.EXACT("Sexual Orientation") OR SU.EXACT("Sex Discrimination") OR Ti,ab("sexual orientation" OR "sexual minority" OR "sexual minorities" OR "sexual orientation" OR homosexual OR homosexuals OR homosexuals' OR gay OR lesbian OR lesbians OR lesbians' OR LGBT OR LGBQ OR transgender OR transgenders OR bisexual OR bisexuals OR bisexuals'))	
Filter: Publication year (1997-2017), scholarly journals	
**Total records #2**	2103
**#1 AND #2)**	6146
