# Supplementary material for: Languishing in the crossroad? A scoping review of intersectional inequalities in mental health
Source: Int J Equity Health. 2019 Jul 24;18:115. doi: 10.1186/s12939-019-1012-4 (PMC6657170; doi:10.1186/s12939-019-1012-4)
Supplement: Supplementary file 2 — Quality Appraisal checklist. Items for quality appraisal and final results. (DOCX 29 kb) [file 12939_2019_1012_MOESM2_ESM.docx]

Quality Appraisal Checklist

1. Study design

The study is preferably primarily designed to analyze intersectional inequalities (e.g. stated as aim or objective and not included as post hoc analysis).

1. Data collection

The study population is randomly selected and preferably representable of the general population. The collection of data on background characteristics such as income and social class is preferably based on registers, and personal characteristics such as sexual orientation is based on validated questions or questionnaires.

1. Outcome measures

Validated scales or diagnostic tools should be used to measure the outcome.

1. Statistical methods

Preferably statistical methods yielding results corresponding to the intersectional inequalities described in (29) are applied.

1. Reporting of intersectional inequalities

Preferably, all results, not only significant estimates, are reported.

| **Study** | **1 study design** | **2 data collection** | **3 outcome measure** | **4 statistical methods** | **5 reporting of results** | **Total score** |
| --- | --- | --- | --- | --- | --- | --- |
| 1. Becker, M., Cortina, K. S., Tsai, Y.-M. & Eccles, J. S. 2014. Sexual orientation, psychological well-being, and mental health: A longitudinal analysis from adolescence to young adulthood. *Psychology of Sexual Orientation and Gender Diversity,* 1**,** 132-145. | 1 | 1 | 1 | 1 | 1 | 5/5 |
| 1. Cohen, J. M., Blasey, C., Barr Taylor, C., Weiss, B. J. & Newman, M. G. 2016. Anxiety and Related Disorders and Concealment in Sexual Minority Young Adults. *Behav Ther,* 47**,** 91-101. | 1 | 1 | 1 | 1 | 1 | 5/5 |
| 1. Davids, C. M. & Green, M. A. 2011. A preliminary investigation of body dissatisfaction and eating disorder symptomatology with bisexual individuals. *Sex Roles,* 65**,** 533-547. | 1 | 1 | 1 | 1 | 0 | 4/5 |
| 1. Evans CR, Williams DR, Onnela J-P, Subramanian S. A multilevel approach to modeling health inequalities at the intersection of multiple social identities. *Social Science & Medicine*. 2018;203:64-73. | 1 | 1 | 1 | 1 | 1 | 5/5 |
| 1. Garratt, E. A., Chandola, T., Purdam, K. & Wood, A. M. 2016. The interactive role of income (material position) and income rank (psychosocial position) in psychological distress: a 9-year longitudinal study of 30,000 UK parents. *Soc Psychiatry Psychiatr Epidemiol,* 51**,** 1361-1372. | 1 | 1 | 1 | 1 | 1 | 5/5 |
| 1. Gibson PA, Baker EH, Milner AN. The role of sex, gender, and education on depressive symptoms among young adults in the United States. Journal of affective disorders. 2016;189:306-13. | 0 | 1 | 1 | 1 | 1 | 4/5 |
| 1. Green, M. J. & Benzeval, M. 2011. Ageing, social class and common mental disorders: Longitudinal evidence from three cohorts in the West of Scotland. *Psychological Medicine,* 41**,** 565-574. | 0 | 1 | 1 | 1 | 1 anxiety, | 4/5 |
|  |  |  |  |  | 0 depression | 3/5 |
| 1. Green, M. J., Espie, C. A. & Benzeval, M. 2014. Social class and gender patterning of insomnia symptoms and psychiatric distress: A 20-year prospective cohort study. *BMC Psychiatry,* 14**,** 9. | 0 | 1 | 1 | 1 | 1 | 4/5 |
| 1. Gustafsson, P. E., Sebastian, M. S. & Mosquera, P. A. 2016. Meddling with middle modalities: a decomposition approach to mental health inequalities between intersectional gender and economic middle groups in northern Sweden. *Glob Health Action,* 9**,** 32819. | 1 | 1 | 1 | 1 | 1 | 5/5 |
| 1. Hardeman, R. R., Przedworski, J. M., Burke, S. E., Burgess, D. J., Phelan, S. M., Dovidio, J. F., Nelson, D., Rockwood, T. & van Ryn, M. 2015. Mental Well-Being in First Year Medical Students: A Comparison by Race and Gender: A Report from the Medical Student CHANGE Study. *J Racial Ethn Health Disparities,* 2**,** 403-13. | 1 | 1 | 1 | 1 | 1 | 5/5 |
| 1. Li, G., Pollitt, A. M. & Russell, S. T. 2016. Depression and Sexual Orientation During Young Adulthood: Diversity Among Sexual Minority Subgroups and the Role of Gender Nonconformity. *Arch Sex Behav,* 45**,** 697-711. | 1 | 1 | 1 | 1 | 1 | 5/5 |
| 1. Lundberg, J., Kristenson, M. & Starrin, B. 2009. Status incongruence revisited: associations with shame and mental wellbeing. *Sociol Health Illn,* 31**,** 478-93. | 1 | 1 | 1 | 1 | 1 | 5/5 |
| 1. Mair, C. A. 2010. Social ties and depression: An intersectional examination of black and white community-dwelling older adults. *Journal of Applied Gerontology,* 29**,** 667-696. | 0 | 1 | 1 | 1 | 1 | 4/5 |
| 1. Rosenfield, S. 2012. Triple jeopardy? Mental health at the intersection of gender, race, and class. *Soc Sci Med,* 74**,** 1791-801. | 1 | 1 | 1 | 1 | 1 | 5/5 |
| 1. Ross CE, Mirowsky J. Sex differences in the effect of education on depression: resource multiplication or resource substitution? *Social science & medicine*. 2006;63(5):1400-13. | 1 | 1 | 1 | 1 | 1 | 5/5 |
| 1. Roxburgh, S. 2009. Untangling inequalities: Gender, race, and socioeconomic differences in depression. *Sociological Forum,* 24**,** 357-381. | 1 | 1 | 1 | 1 | 1 | 5/5 |
| 1. Schieman, S. 2002. Socioeconomic Status, Job Conditions, and Well-being: Self-Concept Explanations for Gender-Contingent Effects. *The Sociological Quarterly,* 43**,** 627-646. | 1 | 1 | 1 | 1 | 1 | 5/5 |
| 1. Strong, S. M., Williamson, D. A., Netemeyer, R. G. & Geer, J. H. 2000. Eating disorder symptoms and concerns about body differ as a function of gender and sexual orientation. *Journal of Social and Clinical Psychology,* 19**,** 240-255. | 1 | 1 | 1 | 1 | 1 | 5/5 |
| 1. Valdez, L. A. & Langellier, B. A. 2015. Racial/Ethnic and Socioeconomic Disparities in Mental Health in Arizona. *Front Public Health,* 3**,** 170. | 0 | 1 | 1 | 1 | 1 | 4/5 |
| 1. Wamala, S., Ahnquist, J. & Månsdotter, A. 2009. How do gender, class and ethnicity interact to determine health status? *Journal of Gender Studies,* 18**,** 115-129. | 1 | 1 | 1 | 1 | 1 | 5/5 |
